# Sexual Orientation, Health, and Well-Being in Spanish People

**DOI:** 10.3390/healthcare12090924

**Published:** 2024-04-30

**Authors:** Roberto Matías, M. Pilar Matud

**Affiliations:** Department of Clinical Psychology, Psychobiology and Methodology, Universidad de La Laguna, 38200 San Cristobal de La Laguna, Spain; alu0101298450@ull.edu.es

**Keywords:** flourishing, gender, homonegativity, life satisfaction, psychological distress, resilience, self-esteem, self-rated health, sexual orientation, social support

## Abstract

Although several studies have found disparities in health outcomes between heterosexual and lesbian, gay, and bisexual (LGB)-identifying individuals, few studies have focused on subjective well-being and protective factors for health and well-being. The purpose of this work is twofold: (1) to examine the relevance of sexual orientation to health and well-being in women and men; (2) to identify protective and risk factors for psychological distress, self-rated health, and well-being for gay men, lesbian women, bisexual women and men, and heterosexual women and men. The sample consisted of 908 women and 586 men from the general Spanish population aged 16–64, half of whom identified themselves as LGB and half as heterosexual. All were assessed using eight questionnaires and inventories. The results showed that differences varied depending on the health indicator considered. In general, bisexuals had the poorest health, with lower self-rated health and lower self-esteem. In all groups, self-esteem was a protective factor against psychological distress and was associated with better health and well-being. To a lesser extent, social support served as a protective factor against psychological distress and was associated with greater well-being in all groups. It is concluded that although sexual orientation is relevant to the health and well-being of individuals, there are differences among sexual minorities, with bisexuals having lower self-esteem than homosexuals.

## 1. Introduction

According to the American Psychological Association [1], sexual orientation “refers to the sex of those to whom one is sexually and romantically attracted” (p. 11). As suggested by this association, typical categories of sexual orientation include attraction to individuals of the same sex (gay men or lesbians), attraction to individuals of the opposite sex (heterosexuals), and attraction to individuals of both sexes (bisexuals). Research has consistently shown that people who identify themselves as lesbian, gay, and bisexual (LGB) have poorer mental health [2,3,4], a higher prevalence of physical health problems [5,6], and poorer self-rated health than heterosexuals [3,7]. However, health disparities between LGB and heterosexual individuals may vary by gender [8,9,10] and other demographic and psychosocial variables [2,3,8].

Although many studies comparing the health of LGB and heterosexual people have considered LGB people as a single group, research has shown that bisexual people are at increased risk for adverse health outcomes compared to homosexual people [2,3,5,11]. It has been suggested that bisexual individuals experience unique stressors, such as negative attitudes toward bisexuality, and challenges related to identity management [12].

The most dominant theory explaining the health disadvantages of LGB people is the minority stress theory [13,14]. Minority stress is an elaboration of social stress theory to distinguish the disproportionate stress experienced by individuals from stigmatized social categories as a result of their social, and frequently minority, position [14]. This theory is based on the conceptualization of stress as external events or conditions that tax and exceed the individual’s ability to endure, recognizing the existence of both individual and social stressors. It is proposed that not only personal events but also social environmental conditions are sources of stress that can lead to physical and mental health problems [14]. Therefore, social stress would be expected to have a strong impact on the lives of people who belong to stigmatized social categories, including gender or sexuality, among others [14]. Thus, this theory posits that LGB people in a heterosexist society are subject to chronic stressors related to their stigmatization. These stressors include internalized homophobia, stigma, and actual experiences of violence and discrimination. According to Meyer [13], internalized homophobia “refers to the direction of social negative attitudes toward the self” (p. 40) and stigma “relates to expectations of rejection and discrimination” (p. 38). And stigma, prejudice, and discrimination create a hostile and stressful social environment that leads to mental health problems [14]. Since chronic exposure to stress is associated with both poorer physical and mental health [14,15,16], the greater exposure of LGB people to stress would explain their poorer health relative to heterosexual individuals. Although stressors strongly influence health, well-being, and behavior, the relationship between psychosocial stressors and disease is influenced by psychosocial resources and coping, as well as the number, type, and persistence of the stressors and an individual’s biological vulnerability [17]. Stress is a complex process [18] in which personal and social resources mediate the effects of stress on health and well-being. In the present work, we consider three resources that we consider particularly relevant: resilience, self-esteem, and social support.

Psychological resilience refers to individuals’ ability to cope positively with life stressors and plays a critical role in helping sexual minority individuals to persist and even flourish despite exposure to stress [19]. Social support is associated with resilience in sexual minorities [20] and has also been identified as a resilience factor for LGB people [19]. Self-esteem is an adaptive trait that has a broad influence on adjustment and adaptation [21]. Self-esteem tends to be lower for sexual minorities than for heterosexual individuals, and these differences may be more pronounced for sexual minority individuals who do not identify themselves as gay or lesbian [22].

Although many studies have examined the relevance of sexual orientation to physical and mental health, fewer have focused on positive aspects and subjective well-being. In addition, most studies have not analyzed data from women and men of different sexual orientations separately. The purpose of this work is twofold: (1) To examine the relevance of sexual orientation to the health and well-being of women and men. (2) To determine the relevance of age, education, homonegativity, fear of negative evaluation, resilience, self-esteem, and social support to psychological distress, self-rated health, and well-being for gay men, lesbian women, bisexual women and men, and heterosexual women and men. Based on the previous literature, the following hypotheses are proposed:

**H1.**  *LGB individuals will experience poorer self-rated health and greater psychological distress than heterosexual individuals.*

**H2.**  *Bisexual individuals will experience poorer self-rated health and lower well-being than homosexual and heterosexual individuals.*

**H3.**  *Resilience will protect against psychological distress and will be associated with better health and greater well-being in LGB and heterosexual individuals.*

**H4.**  *Self-esteem will protect against psychological distress and will be associated with better self-rated health and greater well-being in LGB and heterosexual individuals.*

**H5.**  *Social support will protect against psychological distress and will be associated with better self-rated health and greater well-being in LGB and heterosexual individuals.*

We control that LGB and heterosexual individuals do not differ in age, number of children, marital status, education, and occupation because previous research has shown that LGB and heterosexual individuals differ in sociodemographic characteristics [23]. And there is evidence that such characteristics are relevant to health and well-being [24]. The present study contributes to the literature by analyzing the relevance of major sexual orientations (homosexual, bisexual, and heterosexual) and gender (women, men) to psychological distress, health, and well-being in the population. It also examines the relevance of sociodemographic characteristics such as age and level of education, and psychosocial factors such as homonegativity, fear of negative evaluation, resilience, self-esteem, and social support, on psychological distress, self-reported health, and well-being among people of the different genders and sexual orientations analyzed.

## 2. Materials and Methods

### 2.1. Participants

This is a cross-sectional study conducted with a non-probability sample of 908 women and 586 men aged between 16 and 64 years from the general Spanish population. Half of the sample (*n* = 747) were sexual minorities (homosexual or bisexual), and the other half were heterosexual. The sociodemographic characteristics of age, number of children, education, occupation, and marital status of heterosexuals were controlled for similarity to those of LGB (see Table 1). Although there was diversity in sociodemographic characteristics, the most common was secondary education, which occurred in 57.9% of the total sample, while those with only elementary education were in the minority (5.8%). And just over a third (36.4%) of the sample had studied at university. Almost half of the sample (47.7%) were students, 40.4% were employed, and 10% were unemployed. Most commonly, 69.8% had never been married, 28.3% were married or partnered, and 1.9% were separated or divorced. Most commonly, they had no children, which occurred in 91.9% of the sample. Only 5.3% had one child, 2.1% had two children, and 0.7% had three children.

### 2.2. Procedure

Participation was completely voluntary, and no incentives were offered. Data were collected via an online survey. Participants were electronically sent a link to complete the online survey on their personal computers or on the WhatsApp application, depending on their convenience. After clicking on the survey link, participants received a consent form that explained the content and purpose of the study. If participants consented, the study began with the presentation of a data collection of sociodemographic information and sexual orientation (gay, lesbian, bisexual, heterosexual, other). Participants then completed the psychometric questionnaires and scales described in the Measures subsection. The survey was distributed through various social media platforms and through the researchers’ networks, as well as to undergraduate and graduate university students who were receiving course credit for the task. During data collection, emphasis was placed on sharing the survey link with people of different sexual orientations. In the case of LGB individuals, snowball sampling was also used to get LGB individuals to share the survey link with other homosexual or bisexual individuals. In addition, to increase access to LGB people, contact was made with the *Federación Estatal de Lesbianas, Gays, Trans, Bisexuales, Intersexuales (FELGTBI+)*, the most representative association of sexual minority associations in Spain. It is an organization that includes more than 50 LGTBI+ organizations from all over Spain. The purpose of the study was explained and we were put in contact with FELGTBI+ member organizations. This contact allowed the survey to be distributed both by email and WhatsApp to people from different associations in all of Spain’s Autonomous Communities. The only criterion to participate in the study was to be at least 16 years old.

A total of 2420 people responded to the survey. More than half of them (*n* = 1623; 67.1%) reported being heterosexual, whereas only 29 individuals (1.2%) reported the category “other” (e.g., asexual, pansexual, transsexual, queer, or uncertain). After removing the participants who did not complete the questionnaires and those who responded to the “other” category, the sociodemographic characteristics of the 747 individuals (454 women and 293 men) who reported being homosexual or bisexual were analyzed. Once these characteristics were known, 454 women and 293 men were selected from the sample of 1623 heterosexuals, controlling for the fact that there were no statistically significant differences in age, number of children, marital status, educational level, or occupation with respect to the sexual minority group. The 1975 Declaration of Helsinki, as revised in 2013, was followed in the study. No participant identification data were recorded, and each participant had the right to withdraw from the study at any time. The study received ethical approval from the Research Ethics and Animal Welfare Committee of the University of La Laguna.

### 2.3. Measures

Psychological distress. The Spanish version of the 12-item Goldberg General Health Questionnaire [25] was used to measure participants’ psychological distress. The GHQ-12 is a brief screening instrument and is a widely used measure of psychological distress [26]. Examples of items include “Felt constantly under strain”, “Been feeling unhappy and depressed”, and “Been able to face up to your problems”. Items were scored using the Likert method, which assigns a weight to each score from 0 to 3 (with 0 indicating no distress or reduced functioning). Higher scores indicate greater psychological distress. For the current sample, the internal consistency (Cronbach’s alpha reliability) was high (α = 0.90).

Self-rated general health. This is a single-item ordinal measure that has been widely used as a marker of general health in the general population and is a valid measure of overall health [27,28,29]. In the present study, the response scale was a 5-point scale: very poor, poor, acceptable, good, or very good. Higher values indicated better health.

Life satisfaction. The Satisfaction with Life Scale (SWLS) [30] was used to assess participants’ satisfaction with life. The SWLS consists of five items designed to assess a person’s overall judgment of his/her life satisfaction, which is considered to be the cognitive component of subjective well-being. Examples of items include “In most ways my life is close to ideal”, “The conditions of my life are excellent”, and “I am satisfied with my life”. The items are scored on a 7-point scale, with higher scores indicating greater life satisfaction. For the current sample, the internal consistency was good (α = 0.88).

Flourishing. We used the Flourishing Scale [31], which assesses important aspects of positive psychosocial functioning across multiple domains. It consists of 8 items that describe aspects of human functioning, including feelings of competence, positive relationships, and having meaning and purpose in life. Also included are items on social relationships such as having comforting relationships, being respected by others, and contributing to their happiness, and an item on feeling competent and capable in activities that are important to the respondent [31]. Examples of items include “I am competent and capable in the activities that are important to me”, “I lead a purposeful and meaningful life”, and “I am a good person and live a good life”. Each item is answered on a 7-point scale ranging from strongly disagree to strongly agree, and high scores indicate that the person views himself or herself positively in several important areas. Internal consistency was good for the current sample (α = 0.89).

Self-esteem. The Rosenberg Self-Esteem Scale (RSES) [32] was used to measure participants’ self-esteem. It consists of ten items designed to assess global self-esteem (e.g., “On the whole, I am satisfied with myself”, “I feel that I have a number of good qualities”). The RSES has been validated in many countries and is a widely used instrument for assessing self-esteem [33]. The response scale is a four-point scale, with higher scores indicating greater self-esteem. Internal consistency was good for the current sample (α = 0.89).

Resilience. The Brief Resilience Scale (BRS) [34] was used to assess participants’ resilience. The BRS consists of six items that assess the ability to recover or bounce back from stress (e.g., “I tend to bounce back quickly after hard times”, “It does not take me long to recover from a stressful event”). The items are answered on a five-point scale from strongly disagree to strongly agree, with higher scores indicating greater resilience. For the current sample, the Cronbach’s alpha coefficient was 0.82.

Social support. The Social Support Scale (AS) [35] was used to measure perceived social support. The AS consists of 12 items measuring perceived emotional (e.g., “Someone who comforts you when you are upset”), instrumental (e.g., “Someone who lends you money when you have economic problems”), and informational (e.g., “Someone that gives you information or advice to resolve a problem”) social support. The response scale is a 4-point scale, and higher scores indicate greater perceived social support. The internal consistency across all the items was very high (α = 0.94).

Fear of negative evaluation. We used the Brief Version of the Fear of Negative Evaluation Scale—Straightforward Items (BFNE-S) [36]. It consists of 8 items that measure the extent to which an individual experiences fear of negative evaluation and an expectation of being negatively evaluated. Examples of items include “I am frequently afraid of other people noticing my shortcomings”, “I am afraid that other people will find fault with me”, and “I am usually worried about what kind of impression I make”. The response scale is a five-point scale ranging from “not at all characteristic of me” to “extremely characteristic of me”. For the current sample, the internal consistency was very high (α = 0.94).

Homonegativity, a construct that refers to negative cognitions, affects, and behaviors toward homosexual persons [37], was assessed using the Homonegativity Short Form (HSF) [38]. The HSF consists of ten items with a five-point response format ranging from “strongly disagree” to “strongly agree”. Example of items include “Gay and lesbian people make me nervous”, “I wouldn’t want to have gay or lesbian friends”, and “I fear homosexual persons will make sexual advances to me”. For the current sample, the Cronbach’s alpha coefficient was 0.82.

Demographics. Participants provided basic demographic information, including their gender, age, sexual orientation, education, marital status, number of children, and occupation.

### 2.4. Data Analyses

All the statistical analyses were performed using IBM SPSS Statistics for Windows, version 22. General descriptive statistics were calculated to identify the demographic characteristics of the participants. Comparisons between sexual minorities and heterosexuals for age and number of children were calculated using Student’s *t*-tests, as these were quantitative variables, and Pearson’s chi-square test for education, marital status, and occupation, as these were qualitative variables. To determine the relevance of sexual orientation and gender to the study variables, 2 × 2 between-subjects analyses of variance (ANOVAs) were performed. The independent variables were sexual orientation (homosexual, bisexual, heterosexual) and gender (men, women), and the dependent variables were psychological distress in the first ANOVA, self-rated health in the second, life satisfaction in the third, flourishing in the fourth, self-esteem in the fifth, resilience in the sixth, social support in the seventh, fear of negative evaluation in the eighth, and homonegativity in the ninth ANOVA.

To address the second study aim, hierarchical multiple regression analyses were conducted separately for sexual minorities and heterosexuals, and for women and men. The criterion variable was psychological distress in the first regression analysis group, self-rated health in the second, and well-being in the third. The well-being score was obtained by adding the life satisfaction and flourishing scores. In each regression analysis, age and education were included in the first step (Model 1), homonegativity and fear of negative evaluation were added in the second step (Model 2), and resilience, self-esteem, and social support were added in the third step (Model 3).

## 3. Results

**Figure 1:**
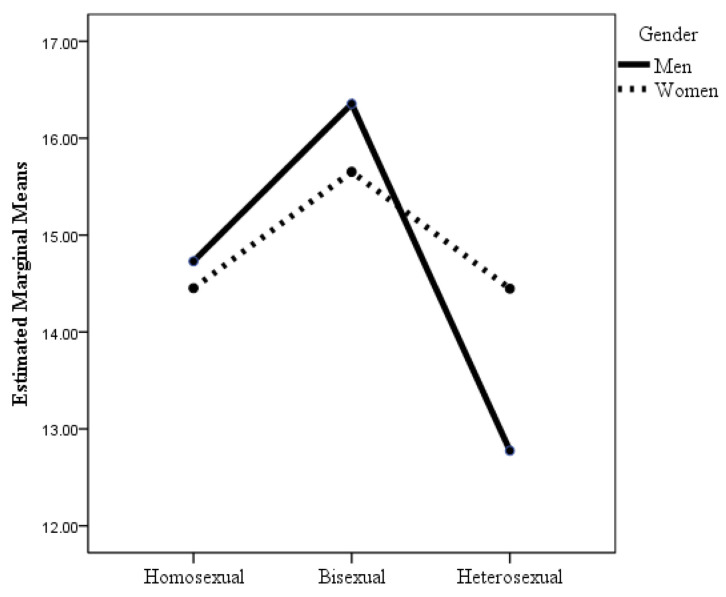
Changes in psychological distress as a function of sexual orientation and gender.

The analysis of the sexual orientation of the 747 people in the sexual minority group revealed that 200 of the men reported being gay, 93 men reported being bisexual, 137 of the women reported being lesbian, and 317 reported being bisexual. Table 2 shows the results of the ANOVAs in which the factors were sexual orientation and gender, and the dependent variables were the indicators of health and well-being considered in this study. When the dependent variable was psychological distress, the interaction effect of sexual orientation × gender was statistically significant (see Table 2 and Figure 1), although the effect size was small. Post hoc analyses with Bonferroni adjustment revealed statistically significant differences between heterosexual men and heterosexual women (*p* = 0.02), gay men (*p* = 0.03), and bisexual women and men (*p* < 0.001). As shown in Table 2 and Figure 1, heterosexual men had lower levels of psychological distress than the other groups, but the differences were not statistically significant (*p* = 0.29) with respect to lesbian women. It highlights that although the mean scores for psychological distress were quite similar for homosexual women and men and for bisexual women and men, and were even slightly higher for men than for women, the opposite was true for heterosexuals. Thus, heterosexual women had more psychological distress than heterosexual men, differences that were statistically significant. However, the mean psychological distress scores for heterosexual women were very similar to those for homosexual women and men, and slightly lower than those for bisexual women and men, although the differences were not statistically significant.

When the dependent variable was self-rated health, only the main effect of sexual orientation was statistically significant, the effect size being small. Post hoc analyses with Bonferroni adjustment revealed statistically significant differences between bisexual women and heterosexual men (*p* = 0.017) and women (*p* = 0.043), with bisexual women reporting worse self-rated health (see Table 2). Bisexual women and men had the lowest mean self-reported health scores. However, the differences between homosexual women and men were not statistically significant. Homosexual women and men also did not differ statistically significantly from heterosexuals in their mean self-reported health scores.

**Figure 2:**
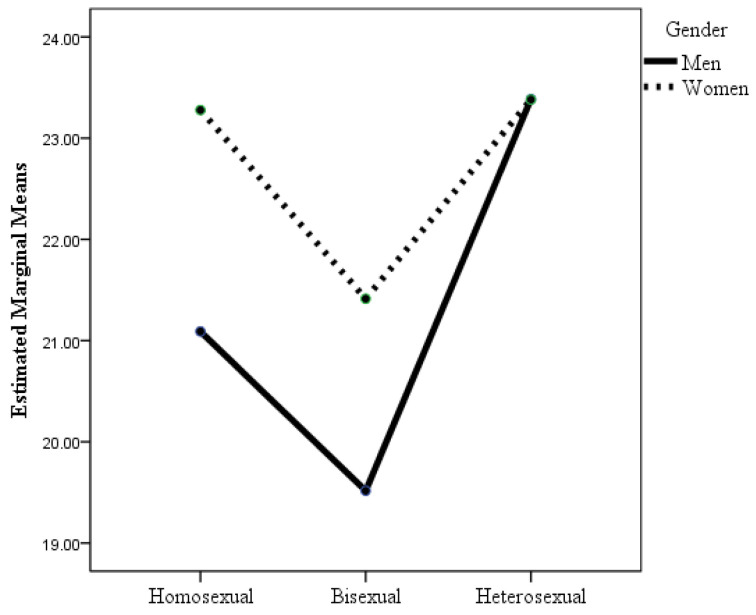
Changes in life satisfaction as a function of sexual orientation and gender.

According to the ANOVA in which the dependent variable was life satisfaction, the sexual orientation × gender interaction was statistically significant (see Table 2 and Figure 2), although the effect size was small. Post hoc analyses with Bonferroni adjustment showed that there were statistically significant differences between gay men and bisexual men and women compared to heterosexual men and women, with gay men and bisexual women and men experiencing lower life satisfaction than heterosexual women and men. In addition, lesbian women had higher life satisfaction than bisexual men (*p* = 0.001). It highlights that while heterosexual women and men were equally satisfied with their lives, this was not the case for sexual minorities, where women were more satisfied with their lives than men, although the differences were not statistically significant. It also highlights that lesbian women’s life satisfaction was about the same as that of heterosexual women and men.

**Figure 3:**
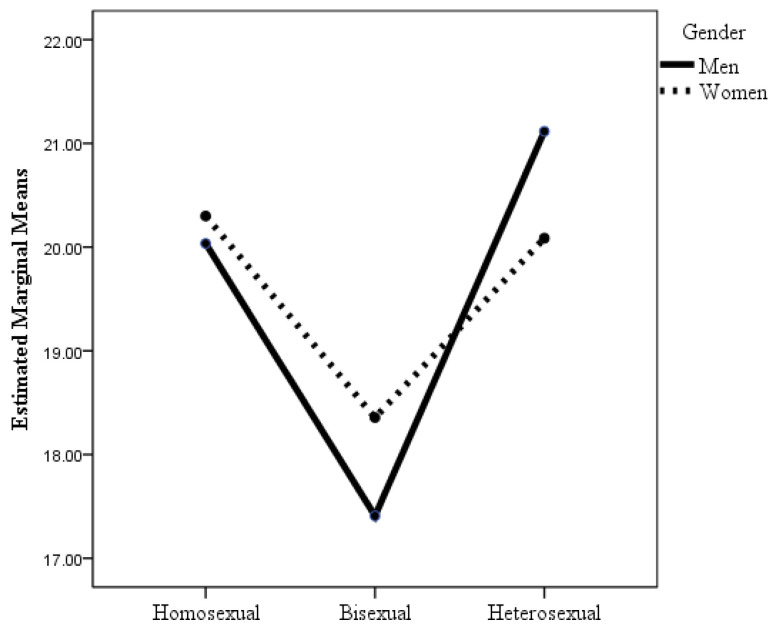
Changes in self-esteem as a function of sexual orientation and gender.

According to the ANOVA with flourishing as the dependent variable, only the main effect of sexual orientation was statistically significant, with the effect size being small. Post hoc analyses with Bonferroni adjustment revealed that statistically significant differences occurred only between bisexual men and heterosexual women, with bisexual men having lower flourishing than heterosexual women (see Table 2). Although the difference in mean scores was very small, especially among heterosexuals, and not statistically significant, it is noteworthy that flourishing was higher among women than among men in all three sexual orientation groups. Of the six groups studied, heterosexual women had the most flourishing, and bisexual men had the least. When the dependent variable was self-esteem, the interaction effect of sexual orientation × gender was statistically significant (see Table 2 and Figure 3), although the effect size was small. Post hoc analyses with Bonferroni adjustment revealed statistically significant differences between bisexual men and women and the other groups, with bisexual women and men reporting lower self-esteem than gay men, lesbian women, and heterosexual women and men.

**Figure 4:**
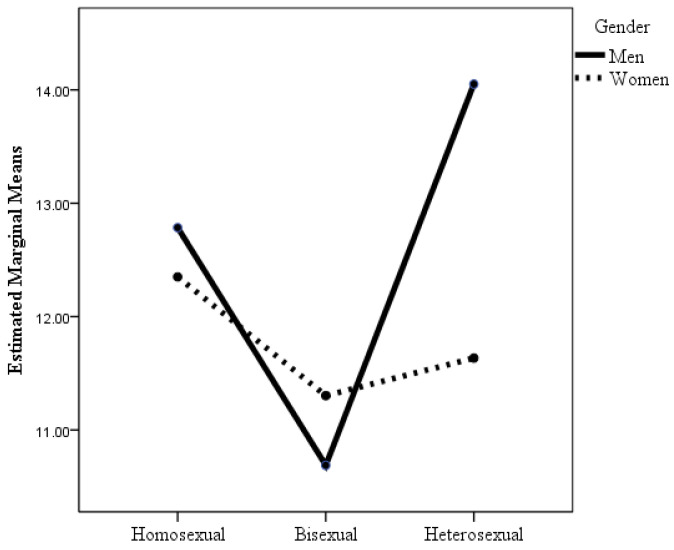
Changes in resilience as a function of sexual orientation and gender.

According to the ANOVA in which the dependent variable was resilience, the interaction between sexual orientation and gender was statistically significant (see Table 2 and Figure 4), although the effect size was small. Post hoc analyses with Bonferroni adjustment revealed that there were statistically significant differences among all groups with respect to heterosexual men and among gay men with respect to all groups except for lesbian women. Heterosexual men were more resilient than the other groups were. Bisexual men were less resilient than the other two groups of men, and the differences were statistically significant. Although bisexual women had lower levels of resilience than the other women, there were no statistically significant differences in resilience among the three groups of women. The resilience of heterosexual and bisexual women was lower than that of heterosexual and homosexual men, but the resilience of lesbian women was lower only relative to heterosexual men.

According to the ANOVA, when the dependent variable was perceived social support, the main effects of gender (*p* < 0.001) and sexual orientation (*p* = 0.001) were statistically significant (see Table 2). The effect sizes for both variables were small. However, the effect size for sexual orientation was much smaller than that for gender. Post hoc analyses with Bonferroni adjustment revealed statistically significant differences between heterosexual women and the three groups of men, between bisexual women and bisexual and gay men, and between lesbian women and bisexual men. Heterosexual women had greater social support than heterosexual, homosexual, and gay men; bisexual women had greater social support than bisexual and gay men; and lesbians had greater social support than bisexual men. Although the differences among women were not statistically significant, heterosexual women had the highest perceived social support, followed by bisexual women and lesbians. And all of the women reported higher levels of social support than the men.

**Figure 5:**
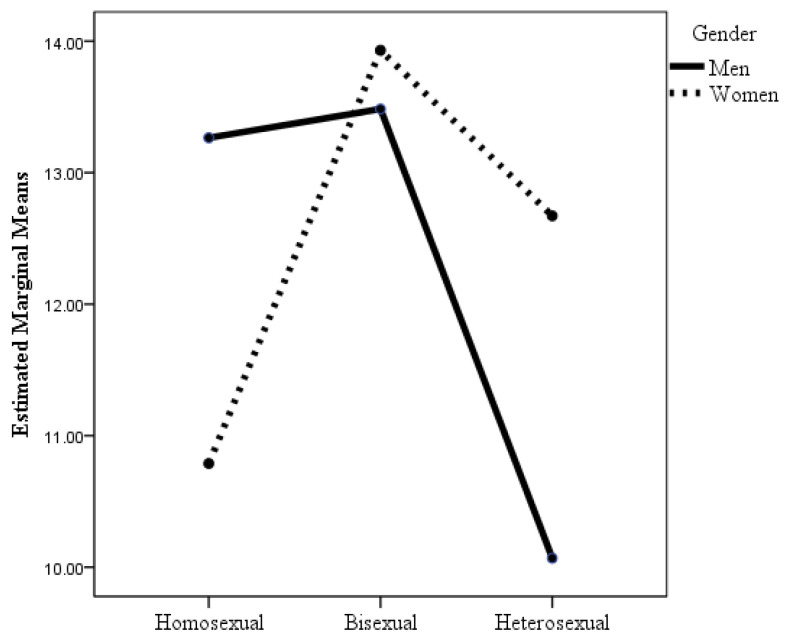
Changes in fear of negative evaluation as a function of sexual orientation and gender.

According to the ANOVA in which the dependent variable was fear of negative evaluation, the interaction effect of sexual orientation × gender was statistically significant (see Table 2 and Figure 5), although the effect size was small. Post hoc analyses with Bonferroni adjustment revealed that there were statistically significant differences between heterosexual men and all the other groups except for lesbian women, and between lesbian women and bisexual women. Bisexual women and men, gay men, and heterosexual women had a greater fear of negative evaluation than heterosexual men did. In addition, lesbian women had less fear of negative evaluation than did bisexual women. There were no statistically significant differences in fear of negative evaluation between lesbian women and heterosexual men.

**Figure 6:**
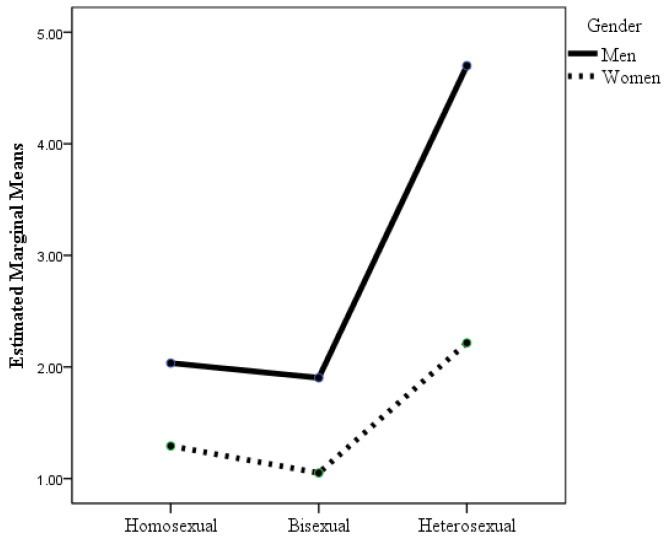
Changes in homonegativity as a function of sexual orientation and gender.

According to the ANOVA where the dependent variable was homonegativity, the interaction effect of sexual orientation × gender was statistically significant (see Table 2 and Figure 6), although the effect size was small. Post hoc analyses with Bonferroni adjustment revealed that there were statistically significant differences (*p* < 0.001) between heterosexual men and the other groups and between heterosexual women and bisexual women. Heterosexual men had greater homonegativity than did bisexual women and men, gay men, lesbian women, and heterosexual women. Although the homonegativity scores of heterosexual women were much lower than those of heterosexual men, their mean scores were somewhat higher than those of the other groups, although the differences were statistically significant only for bisexual women. It highlights that there were no statistically significant differences in homonegativity between homosexuals and bisexuals, although the mean scores of homosexual and bisexual men were slightly higher than those of homosexual and bisexual women.

Table 3 presents the main results of the regression analyses predicting psychological distress in women and men of different sexual orientations. As can be observed in the men’s sample, although younger age was associated with lower psychological distress in bisexual and heterosexual men, age was no longer statistically significant when homonegativity and fear of negative evaluation were added to the equation in Model 2. Adding resilience, self-esteem, and social support to the equation in the third step (Model 3) resulted in an important and statistically significant increase in R^2^ for all three groups of men. According to the final model, with all the variables in the equation, 46% (44% adjusted) of the variability in psychological distress for gay men was predicted, and only two predictors were statistically significant: self-esteem and fear of negative evaluation, with greater psychological distress for gay men with lower self-esteem and greater fear of negative evaluation. For bisexual men, the percentage of variability predicted was 59.4% (56% adjusted), with greater psychological distress for bisexual men with lower self-esteem, lower resilience, and lower homonegativity. For heterosexual men, the percentage of variability predicted was 54.7% (53.5% adjusted), with greater psychological distress for heterosexual men with lower self-esteem, lower resilience, greater fear of negative evaluation, and lower social support.

For the three groups of women, although fear of negative evaluation was statistically significant in Model 2, it was no longer significant when resilience, self-esteem, and social support were added to the equation. According to the final model, with all the variables in the equation, 60% (57.8% adjusted) of the variability in psychological distress was predicted for lesbian women, with those with lower self-esteem and older age experiencing greater psychological distress. The percentage of variability predicted for bisexual women was 46% (44.8% adjusted), with greater psychological distress for those with lower self-esteem and less social support. For heterosexual women, the percentage of variability predicted was 49.3% (48.5% adjusted), with those with lower self-esteem, lower resilience, and lower social support experiencing greater psychological distress.

Table 4 presents the main results of the regression analyses predicting the self-rated health for women and men of different sexual orientations. As can be seen in the men’s sample, although less fear of negative evaluation was a statistically significant predictor of better self-rated health for gay men and heterosexual men, it was no longer statistically significant in Model 3 when resilience, self-esteem, and social support were added to the equation. According to the final model, with all the variables included in the equation, self-esteem was the only statistically significant predictor for gay and bisexual men, with better self-rated health for those with higher self-esteem. For heterosexual men, higher self-esteem also predicted better self-rated health, although to a lesser extent than for the other two groups of men. Other important predictors of better self-rated health for heterosexual men were greater social support, younger age, and greater resilience. The adjusted R^2^ value of 25% indicated that a quarter of the variability in self-rated health was predicted for gay men, a percentage that was lower for heterosexual men (adjusted R^2^ = 19.4%) and for bisexual men (adjusted R^2^ = 14.6%).

In the women’s groups, it is noteworthy that for lesbians and heterosexual women, the sociodemographic variables were statistically significant predictors of better self-rated health in Model 1, although in the final model, with all the variables in the equation, only the beta weight of age was statistically significant. For lesbian women, 29.1% of the variability in self-rated health was predicted, which was greater for those with higher self-esteem, more resilience, and younger age. For bisexual women, the percentage of variability in self-rated health that was predicted was 16.8%, with better self-rated health among those with higher self-esteem. For heterosexual women, the percentage of predicted variability was 18.1%, with better self-rated health among those with higher self-esteem, greater resilience, and younger age.

Table 5 presents the main results of the regression analyses predicting the well-being of women and men of different sexual orientations. As can be observed for gay men, although a higher education and lower homonegativity and fear of negative evaluation predicted greater well-being in Model 2, these variables were no longer statistically significant in Model 3 when resilience, self-esteem, and social support were added to the regression equation. Similarly, for bisexual men, higher education and lower fear of negative evaluation predicted greater well-being in Model 2, but these variables were no longer statistically significant in Model 3 when resilience, self-esteem, and social support were added to the regression equation. The final model showed that gay and bisexual men with higher well-being had higher self-esteem and social support. The percentage of variability in well-being predicted was 62.2% for gay men and 56.2% for bisexual men. For heterosexual men, in addition to higher self-esteem and social support, greater resilience and more education were also statistically significant predictors of greater well-being, predicting 58.5% of the variability in well-being.

In the three groups of women, the R^2^ change values were statistically significant in all three models, although in the final model, the sociodemographic variables were only statistically significant in the group of heterosexual women. Fear of negative evaluation was also statistically significant in all three groups of women in Model 2, although it was no longer significant in Model 3 when resilience, self-esteem, and social support were added to the regression equation. According to the final regression model, with all the variables in the regression equation, greater well-being was associated with greater self-esteem and social support in the three groups of women; in addition, greater well-being was also associated with less homonegativity for lesbian women and with younger age and higher education for heterosexual women. The percentage of predicted variability in well-being predicted was 70.6% for lesbian women, 54.5% for bisexual women, and 53.7% for heterosexual women.

## 4. Discussion

The results of the present study showed the relevance of sexual orientation for the health and well-being of the Spanish general population, in line with findings from other countries [4,5,11,14,39,40,41]. However, although sexual orientation was relevant for all the variables analyzed, the differences depended on the health and well-being indicators considered and, for some of them, on gender. These findings are in line with those of previous research [9,10,29,41,42]. One of the indicators for which fewer differences were found was flourishing, where the effect size of sexual orientation was small, and statistically significant differences were only found between heterosexual women and bisexual men, with greater flourishing among heterosexual women. For self-rated health, the effect size of sexual orientation was small, and statistically significant differences were found only between heterosexual men and women and bisexual women, with bisexual women having worse self-rated health. Although no statistically significant differences in self-rated health were found between heterosexual and bisexual men, this may be due to the fact that the number of bisexual men participating in the study was much lower than that of bisexual women; the mean self-rated health score of bisexual men was slightly lower than that of bisexual women, although their standard deviation was also lower. These findings are only partially consistent with those from other countries where LGB people have been found to be slightly more likely to self-report poorer general health than heterosexual people [3,7,39].

For perceived social support, the effect of gender was larger than that of sexual orientation, although the effect size was small for both variables. Heterosexual women were those who reported the highest social support, which was significantly higher than the social support of men in other groups. The group with the second highest social support was bisexual women, a group with greater social support than bisexual and gay men. In addition, lesbian women reported greater social support than did bisexual men. Statistically significant interactions between sexual orientation and gender were found for the remaining study variables.

Compared to all the other groups, heterosexual men had lower levels of psychological distress, although the differences with lesbian women were not statistically significant. There were also no statistically significant differences in psychological distress between heterosexual women and sexual minorities or between homosexuals and bisexuals. These findings are only partially consistent with those of Liu & Reczek [3], where LGB individuals reported greater psychological distress than heterosexual individuals. In the present study, the lower distress of heterosexual individuals was limited to men. Therefore, the first study hypothesis proposing that LGB individuals would have poorer self-rated health and greater psychological distress than heterosexuals was only partially supported.

There were also no statistically significant differences in fear of negative evaluation between lesbian women and heterosexual men, although heterosexual men was the group with the lowest fear of negative evaluation. In addition, lesbian women had a lower fear of negative evaluation than other women, although the differences were statistically significant only for bisexual women. The fear of negative evaluation of homosexual and bisexual men was very similar and slightly lower than that of bisexual women, although the differences among these three groups were not statistically significant. Although the fear of negative evaluation of heterosexual women was slightly lower than that of these three groups, the differences were not statistically significant. Bisexual men and women and gay men had lower life satisfaction than heterosexual women and men. However, there were no differences in life satisfaction between heterosexuals and lesbian women, who had higher life satisfaction than did bisexual men. Heterosexual men had greater resilience to stress than did the other groups. Gay men had greater resilience than heterosexual women and bisexual men and women. In addition, the self-esteem of bisexual women and men was lower than that of lesbian women, gay men, and heterosexual women and men. Overall, the differential analyses revealed that bisexual individuals have poorer health and less well-being than heterosexual and homosexual individuals, confirming the second study hypothesis. These findings are consistent with those of previous research showing that bisexual individuals report poorer health outcomes in all groups [2,3,5,7,10,22].

The second aim of the current study was to determine the relevance of age, educational level, homonegativity, fear of negative evaluation, resilience, self-esteem and social support for psychological distress, self-rated health, and well-being in gay men, lesbian women, bisexual women and men, and heterosexual women and men. Regression analyses showed that, in all groups, high self-esteem was a factor that protected against psychological distress and promoted health and well-being. Thus, the fourth hypothesis was supported. These results are consistent with those of previous studies that have recognized the relevance of self-esteem for health and well-being [21,43]. Although its relevance was lower, higher social support was also associated with higher well-being in all groups. In addition, higher social support was associated with lower psychological distress in bisexual women, heterosexual men, and heterosexual women and with higher self-rated health in heterosexual men. Thus, the fifth hypothesis was supported, although not fully. These results are consistent with those of previous studies that found better psychological functioning in individuals with greater social support [19,44,45].

Although greater fear of negative evaluation was a statistically significant predictor of greater psychological distress, poorer self-rated health, and lower well-being, its predictive power was no longer statistically significant when resilience, self-esteem, and social support were included in the regression equation. Only in the final model predicting psychological distress for gay men and heterosexual men did greater fear of negative evaluation predict greater psychological distress. The predictive power of resilience also varied by sexual orientation and gender. Greater resilience was associated with lower psychological distress for bisexual men and heterosexual men and women. Greater resilience was also associated with better self-rated health for lesbian women and heterosexual men and women, and with greater well-being for heterosexual men. Therefore, the study’s third hypothesis, which proposed that resilience protects against psychological distress and is associated with greater self-rated health and well-being in LGB and heterosexual individuals, was only partially supported. 

The predictive power of homonegativity was quite limited, but greater homonegativity was associated with lower well-being for lesbian women. Although the reason for the low predictive power of homonegativity in the present study is unknown, it may be a consequence of the fact that homonegativity scores in the sample studied were very low, especially among sexual minorities. More than half of the sample (56%) scored 0 for homonegativity, the median was also 0, and the scores of 83.3% of the sample were less than 4. The maximum score on the scale used was 40 points, but in the sample of the present study the maximum score obtained was 24, which occurred in only three cases. These low levels of homonegativity may be due to the fact that in recent years Spain has made important legislative progress in recognizing the rights of LGBTI people. For example, same-sex marriage was legalized in Spain almost 20 years ago (in July 2005), and Spain is currently one of the leading countries in the recognition of the LGBTI citizens’ rights [46]. Although homophobia persists, its level is low [47]. It has also been shown that Spaniards maintain less social distance and have more contact with homosexuals in terms of quality and quantity than citizens of other countries such as Italy [47]. Nevertheless, LGTBI people in Spain continue to experience discrimination in some areas [46], and homophobia persists. In the present study, heterosexual men reported greater homonegativity than the other groups did, findings that are consistent with those of previous studies [48,49]. However, although the homonegativity of heterosexual women was lower than that of heterosexual men, it was higher than that of bisexual and homosexual women. And although there were no statistically significant differences in homonegativity among sexual minorities, it was higher among men than among women.

The predictive power of age and, especially, educational level for health and well-being was very limited. Nevertheless, it is noteworthy that older age was associated with greater psychological distress for lesbian women, poorer self-rated health for lesbian women and heterosexual women and men, and lower well-being for heterosexual women.

While the current study represents a rigorous investigation in which it is noteworthy that the sociodemographic characteristics of LGB and heterosexual individuals were controlled to be similar, there are limitations that must be considered. First, all of the data were obtained through self-reporting, so there may be biases, particularly social desirability bias. Second, this is a convenience sample, which limits the generalizability of the findings. In addition, the data are cross-sectional, so causal relationships cannot be inferred. Moreover, although the number of women and men in the heterosexual and LGB groups was the same, the results showed that bisexual women were more common in the LGB groups, while the group of bisexual men was significantly smaller. Such differences in group size may affect the finding of statistically significant differences between some groups in the ANOVAs and in the significance of the beta weights in the regression analyses. Finally, other variables that may be relevant, such as cognitive styles or personality variables, were not considered in the prediction of psychological distress and the self-reported health and well-being of LGB and heterosexual individuals. 

Despite these limitations, the results of the present study represent an advance in knowledge of the relevance of sexual orientation and gender to the health of the population. They also represent an advance in the knowledge of the most relevant variables to the health and well-being of women and men of different sexual orientations. These results are useful both for the design of public policies aimed at eradicating homophobia and increasing gender equality, and for educators, psychologists, and other professionals, including those in the media, involved in the design and implementation of educational and psychological intervention programs.

## 5. Conclusions

Although sexual orientation is relevant to the health and well-being of individuals, so is gender, with statistically significant interaction effects between sexual orientation and gender on psychological distress, life satisfaction, self-esteem, resilience, fear of negative evaluation, and homonegativity. Thus, although heterosexual women had greater psychological distress than heterosexual men, there was no difference in psychological distress between LGB women and LGB men. Furthermore, the differences depend on the type of health and well-being indicators involved. Although heterosexual men have lower levels of distress, greater resilience, and less fear of negative evaluation than do the other groups, their homonegativity is also the highest, and on some indicators, they do not differ significantly from lesbian women or gay men. Overall, bisexual women and men have poorer health and self-esteem than heterosexuals and homosexuals. In all groups, self-esteem was a protective factor, reducing the likelihood of psychological distress and being associated with better health and well-being. To a lesser extent, social support served as a protective factor against psychological distress and was associated with greater well-being in all groups. The results of this study have implications for the design of policies and programs to improve the health and well-being of sexual minorities and to improve the health of the general population. The finding that sexual orientation interacts with gender in most of the health and well-being indicators used suggests the relevance of psychosocial factors in population differences to health and well-being. This should be considered in educational programs and public policies aimed at achieving greater equality and well-being for all, especially sexual minorities and women. The relevance of self-esteem as a protective factor against psychological distress and as a factor promoting health and well-being suggests that it is a key variable to be considered in programs and strategies aimed at increasing the well-being of the population, both for sexual minorities and for heterosexuals.

## Figures and Tables

**Table 1:** Demographic characteristics of the lesbian, gay, and bisexual (LGB) and heterosexual people.

	LGB (*n* = 747)		Heterosexual (*n* = 747)		χ^2^
	*n*	%	*n*	%	
Education:					
Elementary	37	5	49	6.6	3.05
Secondary	425	57	439	58.8	
University	284	38.1	259	34.7	
No data	1				
Occupation:					
Student	354	47.8	356	47.7	1.79
Working	294	39.7	307	41.2	
Unemployed	79	10.7	69	9.2	
Retired	10	1.3	12	1.6	
Other	4	0.5	2	0.3	
No data	6		1		
Marital status:					
Never married	513	69.4	524	70.1	0.95
Married/partnered	212	28.7	209	28.0	
Separated/divorced	14	1.9	14	1.9	
No data	8				
	*M*	*SD*	*M*	*SD*	*t*
Age	28.96	10.66	28.28	10.60	1.24
Number of children	0.11	0.44	0.12	0.43	−0.95

**Table 2:** Means (*M*), standard deviations (*SD*), and two-way ANOVA statistics for the study variables.

Variable	Men		Women		ANOVA		
	*M*	*SD*	*M*	*SD*	Effect	*F* Ratio	η^2^_p_
Psychological distress							
Homosexual	14.73	6.74	14.45	7.43	SO	12.44 ***	0.016
Bisexual	16.35	7.23	15.65	7.31	G	0.31	0.000
Heterosexual	12.77	6.64	14.44	6.69	SO × G	4.03 *	0.005
Self-rated health							
Homosexual	2.93	0.82	2.89	0.84	SO	7.15 **	0.010
Bisexual	2.81	0.73	2.83	0.79	G	0.13	0.000
Heterosexual	3.03	0.76	3.00	0.77	SO × G	0.14	0.000
Life satisfaction							
Homosexual	21.09	7.59	23.27	6.90	SO	18.42 ***	0.024
Bisexual	19.52	7.03	21.41	6.99	G	10.85 **	0.007
Heterosexual	23.39	6.54	23.38	6.87	SO × G	3.64 *	0.005
Flourishing							
Homosexual	43.37	8.48	44.03	8.38	SO	6.95 **	0.009
Bisexual	41.99	8.63	43.57	7.57	G	3.24	0.002
Heterosexual	44.61	7.80	44.92	7.66	SO × G	0.66	0.001
Self-esteem							
Homosexual	20.03	5.80	20.29	5.23	SO	24.74 ***	0.032
Bisexual	17.41	6.01	18.36	5.60	G	0.03	0.000
Heterosexual	21.12	5.36	20.09	5.62	SO × G	3.73 *	0.005
Resilience							
Homosexual	12.78	4.81	12.35	4.14	SO	17.32 ***	0.023
Bisexual	10.69	4.28	11.30	4.61	G	7.61 **	0.005
Heterosexual	14.05	4.62	11.63	4.50	SO × G	13.23 ***	0.017
Social support							
Homosexual	24.36	8.71	26.50	8.12	SO	6.96 **	0.009
Bisexual	23.27	8.34	26.89	7.97	G	29.91 ***	0.020
Heterosexual	25.89	7.53	27.85	7.59	SO × G	1.18	0.002
Fear of negative evaluation							
Homosexual	13.26	8.20	10.79	8.15	SO	7.97 ***	0.011
Bisexual	13.48	8.21	13.93	8.85	G	0.15	0.004
Heterosexual	10.07	7.76	12.67	8.60	SO × G	10.39 ***	0.014
Homonegativity							
Homosexual	2.03	3.72	1.29	2.67	SO	36.77 ***	0.047
Bisexual	1.90	3.70	1.05	2.15	G	32.82 ***	0.022
Heterosexual	4.70	5.84	2.22	4.02	SO × G	7.39 ***	0.010

Note. ANOVA = analysis of variance; SO = sexual orientation; G = gender. * *p* < 0.05; ** *p* < 0.01; *** *p* < 0.001.

**Table 3:** Summary of the results of the hierarchical regression for psychological distress for the groups of women and men.

Variable	Gay Men			Bisexual Men			Heterosexual Men		
	Model 1	Model 2	Model 3	Model 1	Model 2	Model 3	Model 1	Model 2	Model 3
	β	β	β	β	β	β	β	β	β
Age	−0.08	0.01	0.10	−0.29 **	−0.17	0.01	−0.12 *	0.05	−0.00
Education	−0.11	−0.13 *	−0.02	−0.15	−0.17	−0.04	−0.05	−0.06	0.06
Homonegativity		0.11	0.03		−0.16	−0.20 *		−0.01	−0.07
Fear of negative evaluation		0.42 ***	0.14 *		0.35 **	−0.07		0.52 ***	0.17 **
Resilience			−0.05			−0.29 **			−0.27 ***
Self-esteem			−0.54 ***			−0.55 ***			−0.40 ***
Social support			−0.09			−0.08			−0.15 **
*R* ^2^	0.018	0.204	0.460	0.116	0.239	0.594	0.018	0.261	0.547
*R*^2^ Change	0.018	0.186 ***	0.256 ***	0.116 **	0.124 **	0.355 ***	0.018	0.243 ***	0.286 ***
	Lesbian women			Bisexual women			Heterosexual women		
	Model 1	Model 2	Model 3	Model 1	Model 2	Model 3	Model 1	Model 2	Model 3
	β	β	β	β	β	β	β	β	β
Age	0.04	0.19 *	0.16 *	−0.13 *	−0.03	0.03	−0.05	0.04	0.07
Education	−0.20 *	−0.21 *	0.04	0.01	0.01	0.07	−0.10 *	−0.06	0.01
Homonegativity		−0.11	−0.09		0.03	0.01		0.01	−0.03
Fear of negative evaluation		0.38 ***	0.08		0.42 ***	0.03		0.40 ***	0.04
Resilience			−0.06			−0.05			−0.21 ***
Self-esteem			−0.67 ***			−0.55 ***			−0.53 ***
Social support			−0.13			−0.18 ***			−0.09 *
*R* ^2^	0.040	0.183	0.600	0.016	0.179	0.460	0.014	0.167	0.493
*R*^2^ Change	0.040	0.143 ***	0.417 ***	0.016	0.163 ***	0.281 ***	0.014 *	0.153 ***	0.326 ***

Note. β = standardized regression coefficient; *R*^2^ = percentage of explained variance; statistically significant β coefficients are shown in bold; * *p* < 0.05; ** *p* < 0.01; *** *p* < 0.001.

**Table 4:** Summary of the results of the hierarchical regression for self-rated health for the groups of women and men.

Variable	Gay Men			Bisexual Men			Heterosexual Men		
	Model 1	Model 2	Model 3	Model 1	Model 2	Model 3	Model 1	Model 2	Model 3
	β	β	β	β	β	β	β	β	β
Age	−0.04	−0.06	−0.12	0.16	0.10	0.04	−0.13 *	−0.23 ***	−0.17 **
Education	−0.02	−0.02	−0.12	0.12	0.12	0.04	0.16 **	0.17 **	0.08
Homonegativity		−0.09	−0.02		0.09	0.13		0.08	0.12 *
Fear of negative evaluation		−0.16 *	0.13		−0.15	0.10		−0.23 ***	−0.01
Resilience			0.11			−0.04			0.14 *
Self-esteem			0.47 ***			0.43 ***			0.22 **
Social support			0.12			0.11			0.17 **
*R* ^2^	0.002	0.036	0.277	0.042	0.067	0.213	0.041	0.098	0.214
*R*^2^ Change	0.002	0.034 *	0.240 ***	0.042	0.025	0.146 **	0.041	0.056 ***	0.116 ***
	Lesbian women			Bisexual women			Heterosexual women		
	Model 1	Model 2	Model 3	Model 1	Model 2	Model 3	Model 1	Model 2	Model 3
	β	β	β	β	β	β	β	β	β
Age	−0.22 *	−0.29 **	−0.26 **	0.00	−0.05	−0.10	−0.14 **	−0.18 **	−0.19 ***
Education	0.18 *	0.18 *	−0.00	0.11	0.11	0.07	0.11 *	0.08	0.03
Homonegativity		0.04	0.01		−0.00	0.01		−0.05	−0.03
Fear of negative evaluation		−0.18 *	0.06		−0.21 ***	0.06		−0.24 ***	−0.02
Resilience			0.20 *			0.03			0.12 *
Self-esteem			0.38 ***			0.41 ***			0.31 ***
Social support			0.13			0.07			0.05
*R* ^2^	0.071	0.101	0.328	0.013	0.055	0.186	0.029	0.085	0.194
*R*^2^ Change	0.071 *	0.030	0.227 ***	0.013	0.042 **	0.131 ***	0.029 **	0.056 ***	0.109 ***

Note. β = standardized regression coefficient; *R*^2^ = percentage of explained variance; statistically significant β coefficients are shown in bold; * *p* < 0.05; ** *p* < 0.01; *** *p* < 0.001.

**Table 5:** Summary of the results of the hierarchical regression for well-being for the groups of women and men.

Variable	Gay Men			Bisexual Men			Heterosexual Men		
	Model 1	Model 2	Model 3	Model 1	Model 2	Model 3	Model 1	Model 2	Model 3
	β	β	β	β	β	β	β	β	β
Age	0.07	0.02	−0.03	0.24 *	0.16	0.07	0.11	0.00	0.07
Education	0.14 *	0.15 *	−0.01	0.16	0.21 *	0.10	0.25 ***	0.25 ***	0.09 *
Homonegativity		−0.21 **	−0.09		−0.13	−0.05		−0.08	−0.02
Fear of negative evaluation		−0.33 ***	0.03		−0.36 ***	0.01		−0.39 ***	0.02
Resilience			0.02			−0.03			0.13 **
Self-esteem			0.59 ***			0.60 ***			0.56 ***
Social support			0.34 ***			0.24 **			0.23 ***
*R* ^2^	0.026	0.181	0.635	0.091	0.242	0.596	0.078	0.215	0.595
*R*^2^ Change	0.026	0.155 ***	0.454 ***	0.091 *	0.151 ***	0.354 ***	0.078 ***	0.137 ***	0.381 ***
	Lesbian women			Bisexual women			Heterosexual women		
	Model 1	Model 2	Model 3	Model 1	Model 2	Model 3	Model 1	Model 2	Model 3
	β	β	β	β	β	β	β	β	β
Age	0.02	−0.09	−0.01	0.09	−0.02	−0.08	−0.01	−0.09	−0.11 **
Education	0.38 ***	0.35 ***	0.07	0.11	0.11 *	0.04	0.19 ***	0.15 **	0.08 *
Homonegativity		−0.13	−0.15 **		0.04	0.08		−0.04	0.01
Fear of negative evaluation		−0.30 ***	0.01		−0.42 ***	−0.01		−0.37 ***	−0.02
Resilience			0.03			−0.00			0.04
Self-esteem			0.61 ***			0.58 ***			0.59 ***
Social support			0.32 ***			0.28 ***			0.20 ***
*R* ^2^	0.147	0.236	0.722	0.024	0.193	0.555	0.035	0.165	0.544
*R*^2^ Change	0.147 ***	0.089 **	0.486 ***	0.024 *	0.169 ***	0.362 ***	0.035 ***	0.130 ***	0.380 ***

Note. β = standardized regression coefficient; *R*^2^ = percentage of explained variance; statistically significant β coefficients are shown in bold; * *p* < 0.05; ** *p* < 0.01; *** *p* < 0.001.

## Data Availability

Data supporting the conclusions drawn in this study are available from the corresponding author if requested.
